# Multimodal Deep Learning-Based Classification of Breast Non-Mass Lesions Using Gray Scale and Color Doppler Ultrasound

**DOI:** 10.3390/diagnostics15232967

**Published:** 2025-11-22

**Authors:** Tianjiao Wang, Qingli Zhu, Tianxiang Yu, Denis Leonov, Xinran Shi, Zhuhuang Zhou, Ke Lv, Mengsu Xiao, Jianchu Li

**Affiliations:** 1 Department of Ultrasound, Peking Union Medical College Hospital, Chinese Academy of Medical Sciences and Peking Union Medical College, Beijing 100730, China; wangtianjiao_19@163.com (T.W.); zqlpumch@126.com (Q.Z.); lvke@163.com (K.L.); 2 Department of Biomedical Engineering, College of Chemistry and Life Science, Beijing University of Technology, Beijing 100124, China; ytx_pedestrian@163.com (T.Y.); shixinran09@163.com (X.S.); zhouzh@bjut.edu.cn (Z.Z.); 3 Research and Education Laboratory, Research and Practical Clinical Center for Diagnostics and Telemedicine Technologies of the Moscow Health Care Department, Moscow 127051, Russia; strat89@mail.ru; 4 Department of Fundamentals of Radio Engineering, Moscow Power Engineering Institute, Moscow 111250, Russia; 5 Department 41, Federal Research Center “Computer Science and Control” of the Russian Academy of Sciences, Moscow 119333, Russia

**Keywords:** breast disease, breast non-mass lesion, ultrasound imaging, artificial intelligence, computer-aided diagnosis

## Abstract

**Objectives:** To propose a multimodal deep learning method for the classification of benign and malignant breast non-mass lesions (NMLs) using grayscale and color Doppler ultrasound and to compare the performance of multi-modality and single-modality breast ultrasound (BUS) models. **Methods:** This retrospective study collected 248 pathologically confirmed NMLs from 241 female patients comprising grayscale and color Doppler BUS images from March 2018 to November 2024. Three types of convolutional neural networks (CNNs), including ResNet50, ResNet18, and VGG16, were evaluated as single-modality (grayscale or color Doppler) models via five-fold cross-validations. The optimal model for each single-modality approach was chosen as the backbone network for multimodal deep learning. Features extracted from grayscale and color Doppler BUS images were then concatenated to predict the probabilities of benignity and malignancy. The diagnostic efficacy of the multi-modality BUS models was comparatively evaluated against single-modality counterparts. **Results:** The single-modality VGG16 models outperformed the other two CNN types for both grayscale and color Doppler BUS using five-fold cross-validations. Additionally, single-modality grayscale models outperformed single-modality color Doppler models. With a mean accuracy of 91.54%, sensitivity of 94.15%, specificity of 87.30%, F1 score of 0.93, and area under the receiver operating characteristic curve (AUC) of 0.96, the multimodal VGG16 models performed better than single-modality counterparts. **Conclusions:** VGG 16-based multimodal ultrasound deep learning showed excellent diagnostic efficacy in distinguishing between benign and malignant NMLs, indicating therapeutic potential to help radiologists assess NMLs.

## 1. Introduction

Breast cancer is the most common cancer in women around the world [1] and early diagnosis through imaging screening reduces the mortality. Breast ultrasound (BUS) has been widely used for detecting and diagnosing breast cancer [2] and can be beneficial in low-resource settings where the availability of mammography is limited [3]. The majority of breast lesions on BUS are three-dimensional masses that are cystic, solid, or mixed [2], which are characterized in the American College of Radiology (ACR) Breast Imaging and Reporting System (BI-RADS^®^). However, there are some breast lesions that are differentiated from normal tissues and lack defined margins and specific shape characteristics of masses. These lesions are thus termed non-mass lesions (NMLs) [2].

Diagnosis of the malignancy of NMLs with BUS remains challenging. NMLs on grayscale BUS lack the three-dimensionality of a mass and are usually characterized by discrete areas of hypoechogenicity and altered echotexture compared to surrounding breast tissues [2,4], which makes the identification of NMLs difficult. Pathologically, NMLs encompass various pathological types, including adenosis, fibrous scars, mastitis, ductal carcinoma in situ, and invasive ductal carcinoma, with benign and malignant lesions exhibiting overlapping ultrasound features such as calcifications and architectural distortions [5,6]. The overlap of these features challenges clinical manual classification of NMLs using BUS, especially given the lack of relevant characterization and risk stratification in the BI-RADS^®^ 5th edition. In addition, manual classification of NMLs on BUS may bring inter- or intra-observer differences.

Artificial intelligence (AI) may provide a complement to clinical diagnosis of NMLs on BUS. AI-based image classification techniques include conventional machine learning and cutting-edge deep learning methods. Previous studies have demonstrated that single-modality grayscale [7] and multi-modality BUS [8]-based machine learning methods are feasible for the NML classification. However, these machine learning methods relied on hand-crafted image feature extraction with specific feature selection. In contrast, deep learning can automatically extract abundant image features with deep neural networks such as convolutional neural networks (CNNs). A previous study introduced a single-modality grayscale BUS deep learning method for NML classification [9], but this approach was limited by the diagnostic information available in single-modality images.

The purpose of this study was to propose a multimodal deep learning method for automatic NML classification based on grayscale and color Doppler BUS and to compare the performance of multi-modality and single-modality BUS-based CNN models.

## 2. Materials and Methods

### 2.1. Patients and Ultrasound Examination

This retrospective study was approved by the Institutional Review Board (IRB) of the Peking Union Medical College Hospital (No. K24C2359), and written informed consent was waived. All procedures were conducted in accordance with the principles of the Declaration of Helsinki. The retrospective study consecutively enrolled 241 female patients with 248 NMLs who underwent ultrasound examinations at the Department of Ultrasound, Peking Union Medical College Hospital, from March 2018 to November 2024. The inclusion criteria were as follows: (a) lesions confirmed as NMLs based on BUS by two expert radiologists according to the Japan Association of Breast and Thyroid Sonology guidelines (T.W. and M.X.), and in cases of disagreement, a third senior radiologist (Q.Z.) reviewed the images, and the final classification was determined by majority consensus; (b) lesions with both grayscale and color Doppler ultrasound images; (c) lesions with definite pathological results based on surgical resection or needle biopsy. The exclusion criteria were as follows: (a) prior surgery or adjuvant therapy; (b) poor ultrasound image quality that impaired the diagnostic interpretation of lesion features.

The ultrasound scanning with both grayscale and color Doppler ultrasound was performed by two expert sonographers (M.X. and Q.Z.), with a clinical ultrasound report generated for each patient. Every breast ultrasound examination was performed following a standardized imaging protocol. Each breast was scanned in multiple planes to guarantee full coverage of all quadrants. For each NML, one grayscale image and one color Doppler image were selected to construct the NML BUS dataset. The grayscale image was chosen as the one that best demonstrated typical morphological characteristics, such as margin definition, shape, and microcalcification, and preferably showed the maximal diameter of the lesion. And the color Doppler image was selected based on the most prominent vascularity within or around the lesion, preferably showing the characteristic blood flow pattern. The BUS images were exported from the ultrasound equipment as JPEG images. All images were reviewed and selected by two experienced radiologists (M.X. and Q.Z.), and any disagreements were settled by consensus to ensure consistency and objectivity.

For equipment settings, there were five ultrasound scanners involved: Philips Affiniti 70 with L12-5/eL18-4/L18-5 linear-array probes (LAPs) [Philips Healthcare, Andover, MA, USA], Siemens Acuson S2000 with the 18L6 HD LAP [Siemens Healthineers, Forchheim, Siemensstraße 3, Germany], Samsung WS80A with the L3-12A LAP [Samsung Healthcare, Suwon, 129, Samsung, Yeongtong, Korea], Supersonic Aixplorer with the SL15-4 LAP [Supersonic Imagine, Aix-en, Provence, France], and Mindary Resona A20 with the LM18-5WU LAP [Mindray Bio-Medical Electronics Co., Ltd., Shenzhen, China]. And 87.9% of NMLs were scanned using the Philips Affiniti 70 system. During scanning, the built-in breast imaging preset was applied to optimize image acquisition. Grayscale parameters such as gain, time-gain compensation (TGC), dynamic range, depth, and focus were adjusted as needed to improve lesion visibility. And color Doppler parameters, including pulse repetition frequency (PRF), wall filter, color gain, and color box size, were optimized to detect low-velocity flow while minimizing noise and artifacts. These standardized settings ensured consistent and high-quality images for subsequent analysis.

### 2.2. NML BUS Dataset Construction and Image Preprocessing

As one representative grayscale image and one color Doppler image were selected for each NML, the dataset comprised 248 NMLs with 248 grayscale and 248 color Doppler BUS images, using pathological results as the reference standard. The size of each image was 1024 pixels (width *W*) × 768 pixels (height *H*). The number of channels (*N*_ch_) of each image was 3.

**Figure 1:**
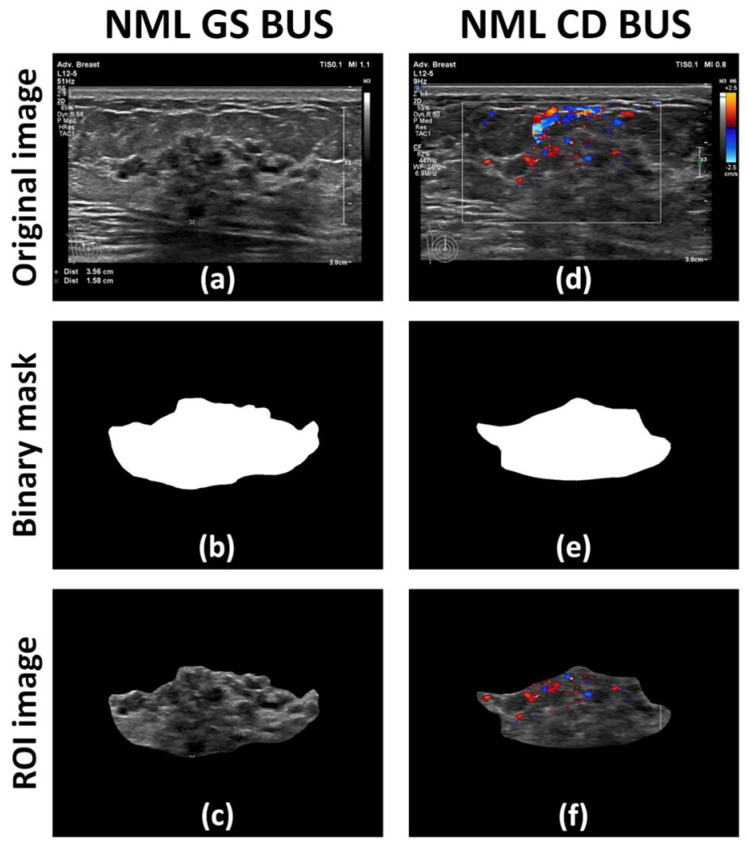
Preprocessing of non-mass lesions (NMLs) ultrasound image: (**a**) and (**d**) are the original gray scale (GS) and color Doppler (CD) images, respectively; (**b**) and (**e**) are the binary mask images based on manual delineation of the NML region of interest (ROI), corresponding to (**a**) and (**d**), respectively; (**c**) and (**f**) are the NML ROI GS and CD BUS images, respectively. This case is an NML of a 31-year-old female patient at the time of ultrasound scanning, and the histopathology result is intraductal papillary carcinoma.

For each original grayscale (Figure 1a) and color Doppler (Figure 1d) BUS image of an NML, a region of interest (ROI) was manually delineated by an experienced radiologist (M.X.) and subsequently confirmed by a senior radiologist (Q.Z.), using the publicly available software LabelMe (version 4.5.13) to generate corresponding binary mask images (Figure 1b,e). In the binary mask image, the values of pixels in the NML region were normalized to 1, and those out of the NML region were normalized to 0. Then, the ROI image of grayscale (Figure 1c) or color Doppler (Figure 1f) BUS was obtained by multiplying the original BUS image by its corresponding binary mask image.

Both ROI grayscale and color Doppler BUS images were resized to 256 pixels (width) × 256 pixels (height) by bicubic interpolation. This algorithm utilizes a cubic convolution kernel based on 16 pixels (4 × 4) around the target pixel to calculate the value of each pixel, thereby achieving image scaling. It performs exceptionally well in maintaining a good balance between smoothness and detail retention. The resized ROI images were used as inputs for the deep neural networks for training and testing the classification models, with each input having a size of 3 × 256 × 256 (*N*_ch_ × *W* × *H*). No data augmentation was conducted.

### 2.3. Partition of Training and Testing Sets

Due to the limited sample size, five-fold cross-validation was performed. Benign and malignant cases were randomly and approximately equally divided into five groups: four with 19 benign and 31 malignant cases each, and one with 18 benign and 30 malignant cases. The data were split in a patient-wise manner, ensuring that all images from the same patient were assigned to the same fold and thus preventing data leakage. Each fold used one group for testing and the rest for training, rotating until all groups were tested.

### 2.4. Single-Modality Deep Learning-Based NML Classification

We first investigated the feasibility of single-modality BUS deep learning in NML classification, using either grayscale or color Doppler ROI images to train and test CNN models. Three types of CNNs, ResNet50, ResNet18, and VGG16, were considered as the deep neural networks for single-modality NML classification because of their promising performance in ultrasound image classification [10,11,12,13].

**Figure 2:**
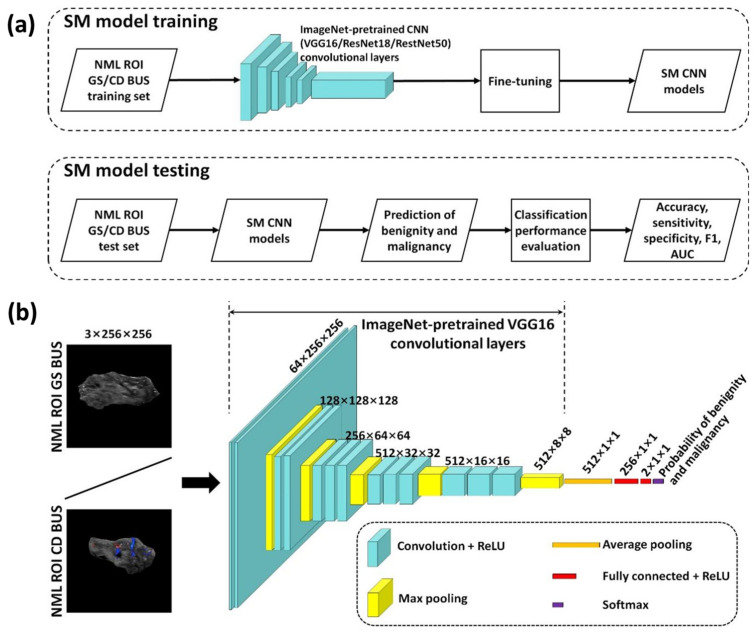
Schematic diagram of single-modality (SM) deep learning-based non-mass lesion (NML) classification: (**a**) presents the training and testing flowchart for SM deep learning models; (**b**) shows the network architecture of SM deep learning models, illustrated using VGG16 models as an example. ROI: region of interest; GS: grayscale; CD: color Doppler; CNN: convolutional neural network; AUC: area under the curve; ReLU: rectified linear unit.

Figure 2a illustrates the workflow of single-modality deep learning for NML classification. For each fold of five-fold cross-validations, VGG16, ResNet50, and ResNet18 single-modality models were trained separately, and the trained models were then used to predict the benign or malignant status of NMLs in the test set. Correspondingly, the diagnostic performance of each model was calculated.

Figure 2b shows the network architecture of single-modality deep learning model. Due to the small sample size in this study, a transfer learning strategy was adopted to mitigate over-fitting of the network. The ImageNet-1K dataset [14] was used to pretrain CNN models, and the network parameters were fine-tuned by the training set of grayscale or color Doppler ROI images. The ImageNet-1K-pretrained CNN models were made publicly available by Torchvison (https://pytorch.org/ accessed on 1 May 2025) and each pretrained model was originally designed for the classification of 1000 categories of nature images. Taking VGG16 model as an example, the convolutional layers of the ImageNet-1K-pretrained CNN model comprised five convolutional modules connected by max pooling layers. The first two modules contained two convolutional layers each, and the last three contained three convolutional layers each, all employing 3 × 3 kernels. Convolutional layers extracted image features into multi-channel feature maps, while pooling layers performed feature compression. Through this process, the original input of 3 × 256 × 256 was transformed into a 512 × 8 × 8 (*N*_ch_ × *W* × *H*) feature map. And the feature map sizes produced by convolutional layers for other models were 2048 × 1 × 1 for ResNet50 and 512 × 1 × 1 for ResNet18. Then, for VGG16, an average pooling layer was added, followed by two fully connected layers with ReLU activations (256 × 1 × 1 and 2 × 1 × 1). For ResNet50 and ResNet18, two fully connected layers with ReLU (512 × 1 × 1 and 2 × 1 × 1 for ResNet50; 256 × 1 × 1 and 2 × 1 × 1 for ResNet18) were added. During this process, the compressed feature maps from the pooling layers were transformed by the fully connected layers into class logits. Subsequently, the softmax layer normalized the logits to yield benign and malignant probabilities for each BUS image, ensuring that the two probabilities summed to 1. Each NML was predicted to be malignant if the probability of malignancy exceeded that of benignity; otherwise, it was predicated to be benign.

The NML classification performance of the three fine-tuned single-modality CNN models (ResNet50, ResNet18, and VGG16) was evaluated and compared using five-fold cross-validation. In each fold of cross-validations, there were six fine-tuned single-modality models: three models trained by the training set of grayscale BUS and three models trained by the training set of color Doppler BUS. Taking the first fold (fold 1) as an example, the six fine-tuned single-modality models were denoted $M_{\mathrm{GS},\mathrm{VGG}16}^{\mathrm{Fold}1}$, $M_{\mathrm{GS},\mathrm{ResNet}50}^{\mathrm{Fold}1}$, $M_{\mathrm{GS},\mathrm{ResNet}18}^{\mathrm{Fold}1}$, $M_{\mathrm{CD},\mathrm{VGG}16}^{\mathrm{Fold}1}$, $M_{\mathrm{CD},\mathrm{ResNet}50}^{\mathrm{Fold}1}$, and $M_{\mathrm{CD},\mathrm{ResNet}18}^{\mathrm{Fold}1}$, where the subscripts “GS” and “CD” represent grayscale and color Doppler, respectively. These experiments showed that the fine-tuned VGG16 models yield the best performance for both grayscale and color Doppler BUS single-modality deep learning (Table 1). Therefore, VGG16 was selected as the backbone network in multimodal deep learning. The network parameters of the convolutional layers of $M_{\mathrm{GS},\mathrm{VGG}16}^{\mathrm{Fold}1}$ to $M_{\mathrm{GS},\mathrm{VGG}16}^{\mathrm{Fold}5}$ and of $M_{\mathrm{CD},\mathrm{VGG}16}^{\mathrm{Fold}1}$ to $M_{\mathrm{CD},\mathrm{VGG}16}^{\mathrm{Fold}5}$ were used to initialize the backbone network in multimodal deep learning for each fold of cross-validations.

### 2.5. Multimodal Deep Learning-Based NML Classification

Based on the findings on single-modality deep learning for NML classification, we investigated multimodal deep learning models to validate the hypothesis that integrating the image features learned by CNNs from dual-modality (grayscale and color Doppler) BUS might improve the classification performance over using single-modality BUS alone.

**Figure 3:**
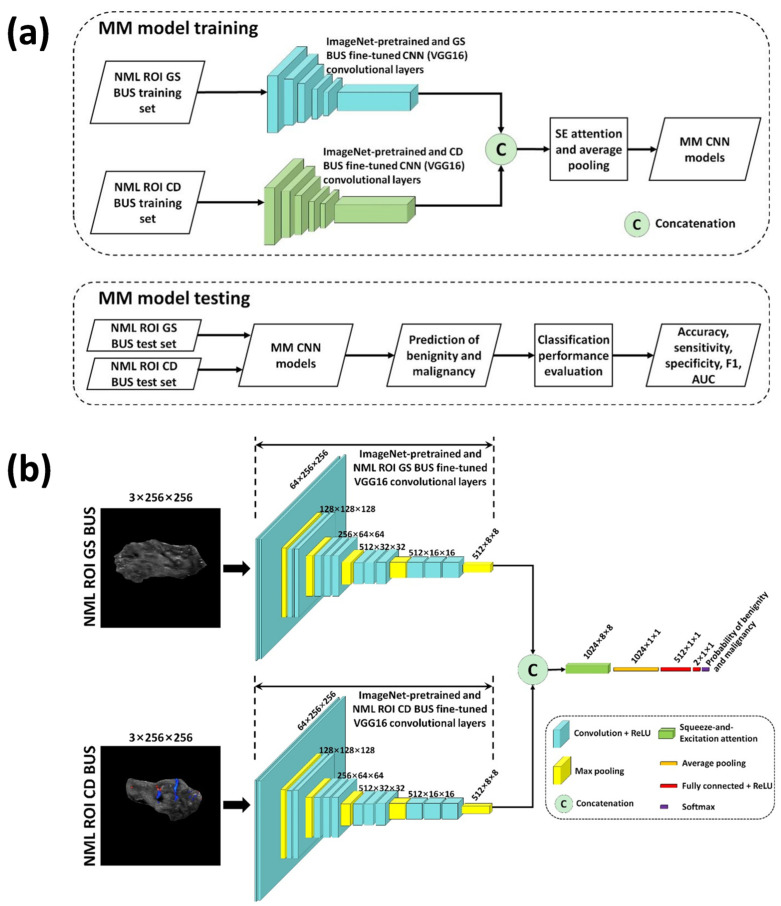
Schematic diagram of multimodal (MM) deep learning-based non-mass lesion (NML) classification: (**a**) presents the training and testing flowchart for MM deep learning models; (**b**) shows the network architecture of MM deep learning VGG16 models. ROI: region of interest; GS: grayscale; CD: color Doppler; CNN: convolutional neural network; SE: Squeeze-and-Excitation; AUC: area under the curve; ReLU: rectified linear unit.

Figure 3a shows the workflow of multimodal deep learning for NML classification. The multimodal CNN models were trained using both grayscale and color Doppler BUS as the inputs. Similar to the single-modality models, the trained multimodal models were applied in each fold of the five-fold cross-validation to predict the benign or malignant status of NMLs in the test set, and the corresponding diagnostic metrics were calculated.

Figure 3b illustrates the network architecture of the multimodal deep learning models. Since the grayscale and color Doppler VGG16 single-modality models showed the best performance in Section 2.4, VGG16 was chosen as the backbone for the multimodal model. To mitigate over-fitting, a transfer learning strategy was also used for multimodal deep learning. We used the network parameters from the convolutional layers of the fine-tuned single-modality VGG16 model described in Section 2.4. Taking the first fold of cross-validations as an example, the network parameters of the convolutional layers of the fine-tuned single-modality VGG16 models $M_{\mathrm{GS},\mathrm{VGG}16}^{\mathrm{Fold}1}$ and $M_{\mathrm{CD},\mathrm{VGG}16}^{\mathrm{Fold}1}$ were used to initialize the two backbone networks of the multimodal CNN model. The sizes of the two feature maps produced by the convolutional layers of $M_{\mathrm{GS},\mathrm{VGG}16}^{\mathrm{Fold}1}$ and $M_{\mathrm{CD},\mathrm{VGG}16}^{\mathrm{Fold}1}$ were both 512 × 8 × 8 (*N*_ch_ × *W* × *H*). Then, the two feature maps were concatenated along the channel dimension using *torch.concat()* function, producing a 1024 × 8 × 8 feature map without requiring image registration or feature alignment. The concatenated feature map was weighted by the Squeeze-and-Excitation attention [15] across the width and height dimensions, followed by one max pooling layer, two fully connected and ReLU layers, and one softmax layer to produce the final benign or malignant prediction for each NML. Let $M_{\mathrm{MM},\mathrm{VGG}16}^{\mathrm{Fold}1}$ denote the multimodal CNN model trained in the first fold of cross-validations, where the subscript “MM” represents multimodal. It should be noted that the training set of $M_{\mathrm{MM},\mathrm{VGG}16}^{\mathrm{Fold}1}$ comprised grayscale BUS images and color Doppler ones, which were the training sets of $M_{\mathrm{GS},\mathrm{VGG}16}^{\mathrm{Fold}1}$ and $M_{\mathrm{CD},\mathrm{VGG}16}^{\mathrm{Fold}1}$, respectively. This also applied to $M_{\mathrm{MM},\mathrm{VGG}16}^{\mathrm{Fold}2}$ to $M_{\mathrm{MM},\mathrm{VGG}16}^{\mathrm{Fold}5}$.

### 2.6. Experimental Setup

All the deep learning models were trained for 30 epochs with batch size of 8. The learning rate was set at 0.001 without any decay schedule. Stochastic gradient descent with momentum (SGDM) was set as the optimizer with momentum of 0.9. Cross entropy was set as the loss function. PyTorch (version 1.12.0) was used as the deep learning framework and Python (version 3.7.9) was used as the programming language. The experiments were performed on a graphics workstation with Intel(R) Xeon(R) Gold 6132 CPU@2.60 GHz 2.59 GHz (2 processors), NVIDIA TITAN RTX 24G GPU, and 128G RAM.

### 2.7. Classification Performance Evaluation

To quantitatively evaluate the NML classification performance by the single-modality and multimodal CNN models, four metrics were used: accuracy, sensitivity, specificity, and F1 score. Receiver operating characteristic (ROC) curve analysis [16] was performed and the area under the curve (AUC) was calculated for each model. The confusion matrices of the multimodal models were also generated.

### 2.8. Statistical Analysis

Categorical variables were expressed as numbers (percentages), normally distributed continuous variables as mean ± standard deviation (SD) with 95% confidence interval (CI), and non-normally distributed variables as median (range). Group comparisons were performed using independent *t* tests for continuous variables and chi-square tests for categorical variables. All analyses were conducted using SPSS Statistics version 25.0 (IBM Corp., Armonk, NY, USA) and Python (version 3.7.9, Python Software Foundation), with *p* < 0.05 considered statistically significant.

## 3. Results

### 3.1. Patient Characteristics

The clinical and pathological characteristics of the study population are presented in Table 2. A total of 248 breast lesions were included, comprising 94 benign and 154 malignant lesions. A significant age difference was observed between the two groups (*p* < 0.001), with patients having malignant lesions (49.9 ± 12.1 years; 95% CI: 48.0–51.9) being older than those with benign lesions (44.6 ± 9.5 years; 95% CI: 42.7–46.6). In terms of clinical symptoms, malignant lesions were more likely associated with palpable masses (*p* = 0.008) and nipple discharge (*p* = 0.015), and benign lesions were more likely asymptomatic and were discovered incidentally through ultrasound examination (*p* = 0.001). The mean ultrasound diameter of NMLs was 2.8 ± 1.6 cm, and malignant NMLs were significantly larger than benign NMLs (3.2 ± 1.7 cm vs. 2.1 ± 1.1 cm, *p* < 0.001). No significant associations were found between malignancy and laterality (*p* = 0.314) or location (*p* = 0.929). The incidence of benign and malignant NMLs in the left and right breasts was almost the same, with the upper outer quadrant being the most common site (benign, 41.5%; malignant, 37.0%). The most common benign NML was glandular disease, accounting for 51.1% of benign cases, and the main malignant NMLs were ductal carcinoma in situ (DCIS) and DCIS with microinvasion, which represented 62.3% of malignancies together.

### 3.2. Performance of Single-Modality and Multimodal CNN Models

Table 1 presents NML classification performance (accuracy, sensitivity, specificity, F1 score, and AUC) by single-modality models for each fold and the mean of five-fold cross-validation. In terms of the mean results of five-fold cross-validations, grayscale BUS-based single-modality models outperformed color Doppler BUS-based ones for each of three single-modality CNNs (ResNet50, ResNet18, and VGG16). Comparing the three types of single-modality CNNs, both grayscale [$M_{\mathrm{GS},\mathrm{VGG}16}^{\mathrm{Fold}1}$ to $M_{\mathrm{GS},\mathrm{VGG}16}^{\mathrm{Fold}5}$]- and color Doppler [$M_{\mathrm{CD},\mathrm{VGG}16}^{\mathrm{Fold}1}$ to $M_{\mathrm{CD},\mathrm{VGG}16}^{\mathrm{Fold}5}$]-based single-modality VGG16 models yield better performance than ResNet50 and ResNet18. Therefore, VGG16 was chosen as the backbone network in multimodal deep learning.

**Figure 4:**
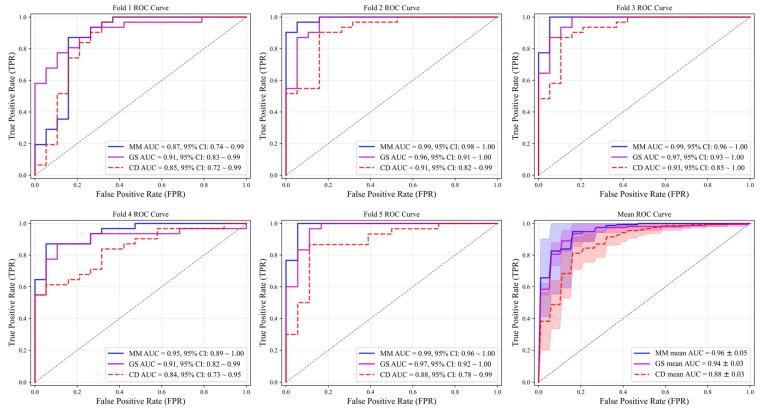
ROC curves for each fold of the five-fold cross-validations of the single-modality and multimodal VGG16 models. Shaded region in the bottom right panel indicates the standard deviation of AUC. MM: multimodal VGG16 model; GS: grayscale ultrasound single-modality VGG16 model; CD: color Doppler ultrasound single-modality VGG16 model; ROC: receiver operating characteristic; AUC: area under the curve.

Table 3 presents NML classification performance (accuracy, sensitivity, specificity, F1 score, and AUC) by multimodal VGG16 models for each fold and the mean of five-fold cross-validation. Table 4 demonstrates the comparison between single-modality and multi-modal models. The multimodal VGG16 models [$M_{\mathrm{MM},\mathrm{VGG}16}^{\mathrm{Fold}1}$ to $M_{\mathrm{MM},\mathrm{VGG}16}^{\mathrm{Fold}5}$] (mean accuracy: 91.54%; mean specificity: 87.30%; mean F1 score: 0.93; mean AUC: 0.96) outperformed the grayscale [$M_{\mathrm{GS},\mathrm{VGG}16}^{\mathrm{Fold}1}$ to $M_{\mathrm{GS},\mathrm{VGG}16}^{\mathrm{Fold}5}$] (mean accuracy: 89.14%; mean specificity: 79.88%; mean F1 score: 0.92; mean AUC: 0.94) and color Doppler [$M_{\mathrm{CD},\mathrm{VGG}16}^{\mathrm{Fold}1}$ to $M_{\mathrm{CD},\mathrm{VGG}16}^{\mathrm{Fold}5}$] (mean accuracy: 82.28%; mean specificity: 86.98%; mean F1 score: 0.86; mean AUC: 0.88) single-modality models in mean accuracy, specificity, F1 score, and AUC. The mean sensitivity of the multimodal VGG16 models (94.15%) was slightly lower than that of the grayscale single-modality VGG16 models (94.81%) but higher than that of the color Doppler single-modality VGG16 models (86.98%). ROC curves for each fold of the five-fold cross-validations of the single-modality and multimodal VGG16 models are plotted in Figure 4. The multimodal model demonstrated superior performance compared with the grayscale model in folds 2–5 and in the mean ROC curve and consistently outperformed color Doppler model across all folds.

**Figure 5:**
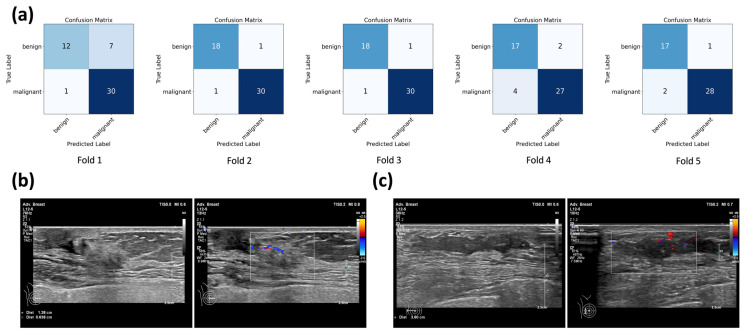
Confusion matrix and misclassification examples of the multimodal VGG16 model: (**a**) shows the confusion matrices of multimodal VGG16 model for each fold in the five-fold cross-validation; (**b**) presents an example of a non-mass lesion (NML) pathologically confirmed as invasive breast carcinoma but misclassified as benign due to poor contrast with the surrounding tissue and limited blood flow; (**c**) presents an example of a NML pathologically confirmed as adenosis but misclassified as malignant due to imaging characteristics including microcalcifications and relatively abundant blood flow.

Figure 5a presents the confusion matrices of the multimodal VGG16 models in distinguishing benign and malignant NMLs. The diagonal elements represent correctly classified cases, while the off-diagonal elements indicate misclassifications. The multimodal VGG16 models showed consistent good classification performance in all five folds, but a few false-positive and false-negative cases were still observed. Some false negatives occurred when malignant NMLs showed poor contrast with the surrounding tissue or exhibited limited blood flow, as illustrated in Figure 5b. In addition, selecting only two representative static images for each lesion may limit the ability of the model to capture all malignant features, which could also contribute to the false-negative predictions. False positives mainly resulted from benign NMLs such as adenosis, whose imaging features were similar to malignant lesions, including microcalcifications and abundant blood flow, as shown in Figure 5c.

## 4. Discussion

This study proposed a novel multimodal deep learning approach combining grayscale and color Doppler BUS for classifying breast NMLs. Three kinds of CNNs including ResNet50, ResNet18, and VGG16 were employed to explore the grayscale single-modality models and color Doppler single-modality models for identifying breast NMLs. Comparative analysis revealed that VGG16 achieved the best performance among the three CNNs in both grayscale and color Doppler single-modality models. Consequently, VGG16 was chosen as the backbone network for the multimodal ultrasound CNN model. In this multimodal CNN model, abundant image features were automatically extracted from grayscale and color Doppler BUS images, respectively, and the extracted features were then fused to predict the malignancy of NMLs. This study demonstrated that the VGG16-based multimodal CNN model outperformed its single-modality counterparts in NML classification, achieving a mean accuracy of 91.54%, mean sensitivity of 94.15%, mean specificity of 87.30%, mean F1 score of 0.93, and mean AUC of 0.96. This study provided a promising tool for assisting in the diagnosis of NMLs.

Radiologists’ understanding of NMLs on ultrasound is still limited, and accurate diagnosis remains challenging, highlighting the potential of AI to assist in NML classification. NMLs include a class of lesions, including benign and malignant lesions, and there is substantial overlap in ultrasound features between malignant and benign non-mass lesions. However, previous studies have demonstrated that segmental distribution, associated calcifications, abnormal duct change, and posterior shadowing were associated with malignancy, whereas multiple small cysts tended to indicate benignity [6,17,18,19]. This suggests that subtle distinctions between benign and malignant ultrasound images can aid in differential diagnosis, providing the basis for the present study. A meta-analysis reported that conventional ultrasound achieved a sensitivity of 93% (95% CI: 82–98%) and a specificity of 52% (95% CI: 33–70%) in the diagnosis of NMLs [20]. These previous studies relied on manual diagnosis, where specificity remains relatively low and requires further improvement. In contrast, the deep learning model developed in the present study can identify imaging characteristics that are not easily recognized through manual visual assessment and extract relevant features automatically. Therefore, the multimodal model achieves higher diagnostic performance.

Grayscale images provide only two-dimensional structural information of NMLs, reflecting morphological characteristics described above, whereas tumor vascularity offers additional diagnostic information that can be captured by color Doppler ultrasound [19,20]. Angiogenesis is closely associated with breast cancer growth, and increased vascularity on color Doppler BUS may indicate malignancy [21]. Previous clinical studies have shown that multi-modality BUS provides more diagnostic value than traditional single-modality grayscale BUS [22,23,24,25,26,27,28,29]. Consistently, the present study found that the multimodal CNN models using grayscale and color Doppler BUS outperformed single-modality CNN models in accuracy, specificity, and AUC, benefiting from the complementary structural information provided by grayscale and vascular information provided by color Doppler. Sensitivity was slightly lower than that of grayscale ultrasound alone, possibly due to the limited sample size. Although additional ultrasound modalities such as elastography or contrast-enhanced ultrasound (CEUS), as well as mammography or breast MRI, were not included, the multimodal model still showed satisfactory diagnostic performance. Given that grayscale and color Doppler BUS are cost-effective and widely available, this multi-modality BUS model may be particularly suitable for regions with limited resources or less advanced technology.

Currently, there are only a few studies on AI for NML classification [7,8,9]. Table 5 compares the proposed multimodal deep learning method with these prior approaches. Shibusawa et al. evaluated the feasibility of a single-modality machine learning-based computer-aided diagnosis (CAD) scheme for improving the performance of clinicians in diagnosing the malignancy of NMLs appearing as hypoechoic areas on grayscale BUS images, and they showed that the AUC increased from 0.65 to 0.78 [7]. Zhang et al. investigated multi-modality BUS-based machine learning for NML diagnosis, where the BUS modalities involved grayscale, color Doppler, strain elastography (SE), and CEUS [8]. Their results showed that the sensitivity and specificity for grayscale, grayscale + color Doppler, grayscale + SE, grayscale + CEUS, and grayscale + color Doppler + SE + CEUS machine learning models were 100% and 29%, 92.5% and 41.9%, 97.5% and 58.1%, 90.0% and 58.1%, and 95.0% and 77.4%, respectively; and the accuracy of these models was 69.0%, 70.4%, 80.2%, 76.1%, and 87.3%, respectively. These findings suggest that combining grayscale with SE and CEUS achieved the best specificity and accuracy and relatively high sensitivity, suggesting that multi-modality BUS might be helpful for diagnosing NMLs [8]. Li et al. investigated single-modality grayscale BUS-based deep learning for NML differentiation using two kinds of CNNs: DenseNet121 and MobileNet [9]. The MobileNet model achieved the higher diagnostic performance in the testing set (without cross-validations), with the AUC, accuracy, sensitivity, and specificity being 0.84, 70.5%, 80.3%, and 74.6%, respectively [9]. However, multi-modality BUS-based deep learning remains underexplored. Therefore, in this study, we used a multimodal deep learning method to classify NML. The average AUC, accuracy, sensitivity, and specificity of the five-fold cross-validations were 0.96, 91.54%, 94.15%, and 87.30%, respectively, which are better than the diagnostic performances reported in previous studies. The application of multimodal deep learning shows great potential to provide more objective and consistent diagnostic results and offer a supplementary diagnostic aid for radiologists to evaluate NML.

Regarding the choice of deep learning models, it is noteworthy that VGG16 outperformed the other two models in this study, which is consistent with prior cardiac ultrasound findings [13]. VGG16 demonstrates considerable potential as a backbone for developing deep learning models aimed at the classification of NMLs.

This study had several limitations. First, this study only used a dataset from a single center and lacked external validation, thus introducing uncertainty regarding its generalizability. And static BUS images may not fully capture all the characteristics of NMLs, which can lead to a certain degree of misclassification. Thus, future studies should establish larger, multi-center datasets that include dynamic video sequences for model training and further evaluate generalizability across diverse datasets from multiple centers and populations. Moreover, as the dataset was collected from tertiary hospitals for diagnostic rather than screening purposes, the application of this model is limited to diagnostic use. Second, most images were acquired using Philips ultrasound systems, although a small number were obtained from other ultrasound systems. This may introduce system-related differences and potential scanner-dependent effects. Future studies should include images from multiple ultrasound systems to further validate and generalize the model. Third, the multimodal method only used the two most common modalities, grayscale and color Doppler BUS, though ultrasound elastography [30,31,32,33,34] and CEUS [35,36] have demonstrated additional diagnostic value. In future research, new ultrasound techniques, such as ultrasound elastography and CEUS, can be integrated, which may further enhance the NML classification performance. Fourth, observed differences in clinical characteristics between patients with malignant and benign lesions indicate that integrating clinical information such as age, clinical symptoms, and lesion size into future models could further improve diagnostic performance. Fifth, although the model showed promising performance, its interpretability remains limited, highlighting the necessity of exploring explainable AI methods for NML feature description. Finally, this study relied on manually delineated ROIs and focused on the classification of NMLs as benign or malignant. However, due to the indistinct boundaries and the lack of a well-defined three-dimensional mass structure, identifying NMLs remains a significant challenge in clinical practice, which warrants further investigation in future studies.

## 5. Conclusions

In conclusion, this study proposed a multimodal deep learning method combining grayscale and color Doppler BUS for NML classification. The multimodal deep learning method demonstrated excellent performance in classification, providing more objective diagnostic outcomes. The new multimodal method shows promise as a clinical decision support tool for radiologists in ultrasound-based NMLs assessment.

## Figures and Tables

**Table 1:** Non-mass lesion (NML) classification performance by single-modality models based on grayscale (GS) or color Doppler (CD) breast ultrasound.

Fold	Accuracy		Sensitivity		Specificity		F1		ROC	
	GS	CD	GS	CD	GS	CD	GS	CD	GS	CD
ResNet50										
1	85.99%	71.99%	93.54%	83.87%	73.68%	52.63%	0.89	0.79	0.94	0.74
2	89.99%	65.99%	96.77%	77.41%	78.94%	47.36%	0.92	0.74	0.95	0.71
3	83.99%	69.99%	83.87%	70.96%	84.21%	68.42%	0.87	0.75	0.95	0.75
4	71.99%	68.00%	67.74%	74.19%	78.94%	57.89%	0.75	0.74	0.82	0.66
5	79.16%	58.33%	76.66%	53.33%	83.33%	66.66%	0.82	0.62	0.93	0.66
Mean	82.23%	66.86%	83.72%	71.95%	79.82%	58.59%	0.85	0.73	0.93	0.70
ResNet18										
1	85.99%	65.99%	96.77%	77.41%	68.42%	47.36%	0.90	0.74	0.94	0.75
2	89.99%	68.00%	93.54%	83.87%	84.21%	42.10%	0.92	0.76	0.96	0.77
3	81.99%	77.99%	77.41%	93.54%	89.47%	52.63%	0.84	0.84	0.93	0.87
4	81.99%	69.99%	87.09%	83.87%	73.68%	47.36%	0.86	0.78	0.86	0.77
5	85.41%	72.91%	83.33%	83.33%	88.88%	55.55%	0.88	0.79	0.96	0.84
Mean	85.08%	70.98%	87.63%	84.40%	80.93%	49.00%	0.88	0.78	0.93	0.80
VGG16										
1	81.99%	79.99%	93.54%	83.87%	63.15%	73.68%	0.87	0.84	0.91	0.85
2	91.99%	83.99%	96.77%	93.54%	84.21%	68.42%	0.94	0.88	0.96	0.91
3	94.00%	85.99%	100.00%	87.09%	84.21%	84.21%	0.95	0.89	0.97	0.93
4	83.99%	75.99%	87.09%	87.09%	78.94%	57.89%	0.87	0.82	0.91	0.84
5	93.75%	85.41%	96.66%	83.33%	88.88%	88.88%	0.95	0.88	0.97	0.88
Mean	89.14%	82.28%	94.81%	86.98%	79.88%	74.61%	0.92	0.86	0.94	0.88

**Table 2:** Clinical and pathological characteristics of non-mass lesions of non-mass lesions (NMLs). CI: confidence interval of the average age; DCIS: ductal carcinoma in situ; UIQ: upper inner quadrant; LIQ: lower inner quadrant; UOQ: upper outer quadrant; LOQ: lower outer quadrant; UOQ–UIQ: superior border between UOQ and UIQ; UOQ–LOQ: lateral border between UOQ and LOQ; LOQ–LIQ: inferior border between LOQ and LIQ; UIQ–LIQ: medial border between UIQ and LIQ; CZ: central zone.

	Benign		Malignant		Total	*p*-Value
Number	94		154		248	
Age	44.6 ± 9.5 (95%CI: 42.7—46.6)		49.9 ± 12.1 (95%CI: 48.0—51.9)		47.9 ± 11.5	<0.001
≤40 years	36 (38.3%)		34 (22.1%)		70 (28.2%)	0.006
>40 years	58 (61.7%)		120 (77.9%)		178 (71.8%)	
Clinical symptoms						
Mass	16 (17.0%)		50 (32.5%)		66 (26.6%)	0.008
Pain	5 (5.3%)		12 (7.8%)		17 (6.9%)	0.455
Nipple discharge	5 (5.3%)		24 (15.6%)		29 (11.7%)	0.015
Nipple retraction	0 (0%)		4 (2.6%)		4 (1.6%)	0.291
Skin redness	1 (1.1%)		4 (2.6%)		5 (2.0%)	0.713
Asymptomatic	68 (72.3%)		78 (50.6%)		146 (58.9%)	0.001
NML size (cm)	2.1 ± 1.1		3.2 ± 1.7		2.8 ± 1.6	<0.001
Laterality						0.314
Right	39 (41.5%)		74 (48.1%)		113 (45.6%)	
Left	55 (58.5%)		80 (51.9%)		135 (54.4%)	
Location						0.929
UIQ	13 (13.8%)		22 (14.3%)		35 (14.1%)	
LIQ	6 (6.4%)		9 (5.8%)		15 (6.0%)	
UOQ	39 (41.5%)		57 (37.0%)		96 (38.7%)	
LOQ	10 (10.6%)		24 (15.6%)		34 (13.7%)	
UOQ-UIQ	12 (12.8%)		16 (10.4%)		28 (11.3%)	
UOQ-LOQ	10 (10.6%)		15 (9.7%)		25 (10.1%)	
LOQ-LIQ	1 (1.1%)		1 (0.6%)		2 (0.8%)	
UIQ–LIQ	2 (2.1%)		6 (3.9%)		8 (3.2%)	
CZ	1 (1.1%)		4 (2.6%)		5 (2.0%)	
Pathological type						
	Glandular disease	48 (51.1%)	DCIS	57 (37.0%)		
	Intraductal papilloma	16 (17.0%)	DCIS with microinvasion	39 (25.3%)		
	Fibroadenoma	15 (16.0%)	Invasive ductal carcinoma	49 (31.8%)		
	Mammary tissue	12 (12.8%)	Lobular carcinoma in situ	1 (0.6%)		
	Fibrocystic disease	2 (2.1%)	Invasive lobular carcinoma	8 (5.2%)		
	Hamartoma	1 (1.1%)				

**Table 3:** Non-mass lesion (NML) classification performance by multimodal (MM) VGG16 models.

Fold	Model	Accuracy	Sensitivity	Specificity	F1	AUC (95%CI)
1	$M_{\mathrm{MM},\mathrm{VGG}16}^{\mathrm{Fold}1}$	83.99%	96.77%	63.15%	0.88	0.87 (0.74–0.99)
2	$M_{\mathrm{MM},\mathrm{VGG}16}^{\mathrm{Fold}2}$	95.99%	96.77%	94.73%	0.97	0.99 (0.98–1.00)
3	$M_{\mathrm{MM},\mathrm{VGG}16}^{\mathrm{Fold}3}$	95.99%	96.77%	94.73%	0.97	0.99 (0.96–1.00)
4	$M_{\mathrm{MM},\mathrm{VGG}16}^{\mathrm{Fold}4}$	88.00%	87.09%	89.47%	0.90	0.95 (0.89–1.00)
5	$M_{\mathrm{MM},\mathrm{VGG}16}^{\mathrm{Fold}5}$	93.75%	93.33%	94.44%	0.95	0.99 (0.96–1.00)
Mean	$M_{\mathrm{MM},\mathrm{VGG}16}^{\mathrm{Fold}1}$ to $M_{\mathrm{MM},\mathrm{VGG}16}^{\mathrm{Fold}5}$	91.54%	94.15%	87.30%	0.93	0.96 ± 0.05

**Table 4:** Comparison of NML classification performance by grayscale (GS) and color Doppler (CD) single-modality and by multimodal (MM) VGG16 models.

Model	Mean Accuracy	Mean Sensitivity	Mean Specificity	Mean F1	Mean AUC
$M_{\mathrm{GS},\mathrm{VGG}16}^{\mathrm{Fold}1}$ to $M_{\mathrm{GS},\mathrm{VGG}16}^{\mathrm{Fold}5}$	89.14%	94.81%	79.88%	0.92	0.94
$M_{\mathrm{CD},\mathrm{VGG}16}^{\mathrm{Fold}1}$ to $M_{\mathrm{CD},\mathrm{VGG}16}^{\mathrm{Fold}5}$	82.28%	86.98%	74.61%	0.86	0.88
$M_{\mathrm{MM},\mathrm{VGG}16}^{\mathrm{Fold}1}$ to $M_{\mathrm{MM},\mathrm{VGG}16}^{\mathrm{Fold}5}$	91.54%	94.15%	87.30%	0.93	0.96

**Table 5:** Comparison of the proposed multimodal (MM) deep learning method with state-of-the-art methods for artificial intelligence-based non-mass lesion (NML) classification on breast ultrasound (BUS); SM: single-modality; ML: machine learning; DL: deep learning; GS: grayscale; CD: color Doppler; SE: strain elastography; CEUS: contrast-enhanced ultrasound; NR: not reported.

Study	Year	Method	Modality	Patients Number	With Cross-Validations	NML Classification Performance			
						AUC	Accuracy	Sensitivity	Specificity
Shibusawa et al. [7]	2016	SM ML	GS	97	No	0.78	NR	NR	NR
Zhang et al. [8]	2018	MM ML	GS + CD + SE + CEUS	71	No	NR	87.3%	95.0%	77.4%
Li et al. [9]	2023	SM DL	GS	228	No	0.84	70.5%	80.3%	74.6%
This study	2025	MM DL	GS + CD	248	Yes	0.96	91.5%	94.2%	87.3%

## Data Availability

The data presented in this study are available on request from the corresponding author (M.X.) due to patient privacy concerns and ethical approval restrictions.

## Abbreviations

The following abbreviations are used in this manuscript:

BUS	Breast ultrasound
ACR	American College of Radiology
BI-RADS^®^	Breast imaging and reporting system
NML	Non-mass lesion
AI	Artificial intelligence
CNN	Convolutional neural network
LAP	Linear-array probe
ROI	Region of interest
ReLU	Rectified linear unit
GS	Grayscale
CD	Color Doppler
MM	Multimodal
SGDM	Stochastic gradient descent with momentum
ROC	Receiver operating characteristic
AUC	Area under the curve
SD	Standard deviation
CI	Confidence interval
CAD	Computer-aided diagnosis
SE	Strain elastography
CEUS	Contrast-enhanced ultrasound
